# A Reconsideration of the Link between the Energetics of Water and of ATP Hydrolysis Energy in the Power Strokes of Molecular Motors in Protein Structures

**DOI:** 10.3390/ijms9091730

**Published:** 2008-09-09

**Authors:** Wilfred F. Widdas

**Affiliations:** Faculty of Science, University of London, UK

**Keywords:** Adenosine Triphosphate (ATP), ATP-splitting, chemical energy, surface energy of water, First Law of Thermodynamics, Joule/Helmholtz concept, Fenn Effect

## Abstract

Mechanical energy from oxygen metabolism by mammalian tissues has been studied since 1837. The production of heat by mechanical work was studied by Fick in about 1860. Prior to Fick’s work, energetics were revised by Joule’s experiments which founded the First Law of Thermodynamics. Fenn in 1923/24 found that frog muscle contractions generated extra heat proportional to the amount of work done in shortening the muscle. This was fully consistent with the Joule, Helmholtz concept used for the First Law of Thermodynamics. The link between the energetics of water and ATP hydrolysis in molecular motors is recommended for reconsideration.

## 1. Introduction

Reviews are of varying types but this article is not so much a looking back or critical reappraisal but essentially the physiological reconsideration in the light of advances in many techniques used in molecular science and biophysics. Many biochemical muscle scientists have used advanced techniques from related disciplines and have made their own interpretations, which I feel need revision. Thus, although no longer active in University research, I am still able to study simple molecules by atomic model building and reading reprints of other investigators. In addition there are some excellent scientific articles on the Internet written by unnamed experts in their fields, to whom I am deeply grateful.

The mechanical energy available from movements can most easily be quantified as force times distance to give a measure of work. The SI unit for work is the Joule, which has replaced the older heat unit (calorie). Movements in mammals are chiefly made by muscles and have been associated with the evolution of heat energy attributed to oxygen metabolism in tissues since 1837. Prior to 1837, the current view was expressed by Lavoisier, who thought that the oxygen was all used by the lungs. It is now agreed that oxygen is used by all tissues and not only by the lungs.

This metabolic energy source has become narrowed down by research to the way the food we eat can be oxidised in a very piecemeal fashion to yield Adenosine Triphosphate hydrolysis as the final currency for the chemical energy available for both heat and mechanical work in the tissues. However, ATP hydrolysis is only one of several energy sources to be considered. The surface energy of water, and of other liquids, forms non-chemical sources of energy that have the ability to do mechanical work in biological systems. The available energy sources for physiological movements and work in mammals are the main topics for discussion and reconsideration in the present review.

ATP hydrolysis has been extensively studied in chemistry and biochemistry. Surface energy has been studied in the physical sciences, such as biophysics or physical chemistry. When mechanical work is done there must be movements created. Thermal vibrations, such as Brownian movements, can be in any direction and are random movements. Movements in specific directions are the properties of free bodies in motion and were recognised by Newton to have momentum (p = mv) in one conserved direction. The kinetic energy of moving bodies is dependent upon both the mass and the square of the velocity (k.e. = ½ mv^2^).

Energetics is defined as the science of the general laws of energy employment. For the present author’s purpose, an important aspect is a fuller understanding of the First Law of Thermodynamics, which was founded by the experimental work in physics completed by Joule in the 1840s and will be discussed in this report.

### 1.1. Energy received from the Sun: sharing of two energy sources

Philosophically, one can argue that life on this planet is supported by radiation from the Sun. Short wave-length energy is used for photosynthesis to produce oxidisable chemicals from CO_2_ and water. The longer wave-length radiation provides the energy that replaces the heat energy lost from the oceans by the latent heat of evaporation of water. This longer radiation from the Sun keeps the global energy in balance as a steady state. The consequences of this energy source were outlined by Widdas and Baker [1].

The energy source for the movements in mammals – such as muscle contraction – is still not completely understood. This review centres its reconsideration on metabolic and non-metabolic energy, rather than on schemes of shortening which assume that all the work is done by one single energy source. That both energy sources may be involved in producing mechanical work in molecular motors and their linkage to make this sharing possible is a theme of the present article.

Movements can only be made by mechanisms that use a source of energy. In addition to the energy source, an engine or engine-like mechanism is required to transfer the energy into a form of mechanical work. In short, there is an energetic problem and this involves understanding the physical chemical principles of the work potential of both the chemical and non-metabolic energy sources. Thus, petrol as a source of chemical energy for a car or boat is useless without an internal combustion engine to convert the chemical energy from oxidative metabolism into meaningful physical movement.

To plan an “engine” and explain how it can work is necessary to gain a reappraisal and reinterpretation of experimental accounts published in the literature on this topic. The advanced techniques of present day physiology include cellular and membrane transport as well as protein contractions. Modern advances in these topics or related sciences are often described on the Internet, in textbooks or as available reprints of published work by relevant authors.

## 2. Results and Discussion

### 2.1. Electrostatic forces: the only important form of energy for movements

There are several forms of mechanical work or movements to be considered in mammalian Physiology. Random thermal agitations in living systems are irrelevant to this report, as it studies movements in many proteins, which need to occur in precisely fixed directions. Directional movements are needed for muscle contraction, the action of kinesin and the replication of cells and cell nuclei genetically.

Physical chemical hypotheses to explain purposeful movements may be limited to electrostatic forces between electric charges. In the present review, therefore, electrostatic forces are regarded as the only forces of importance to muscle and molecular science. Electrostatic forces are based on the original work by Coulomb and can be simply expressed as due to the product of the charges divided by the square of the separating distance times the dielectric constant:

(1) $$\text{Force }=\text{ Constant}\times {\text{q}}_{1}{\text{ q}}_{2}/({\text{d}}^{2}.ɛ)$$

where q_1_ and q_2_ are the two charges, d the separation distance and ɛ the dielectric constant. In MKS units the Force constant for unit electric charges was given by Widdas [1] as 2.3 × 10^−28^ Nm^2^. The dielectric constant of water is 80.

Like charges can cause movements in tissues and act to increase separation, whereas unlike charges produce extra cohesion in their movements. Further, the charges may not be full electronic charges but delta charges as with dipole molecules. Water is regarded as a permanent double dipole and the polar value for each hydrogen atom of the water molecule (H_2_O) has a delta-positive charge that is roughly estimated as 0.26 of a full charge.

In consequence, for neutrality, the oxygen nucleus must act as if it had a delta-negative charge of 0.52 of a full charge. For rough calculations, these delta charges are rounded off and taken to be ¼ and ½ respectively. The product (1/8) is eight times smaller than the forces between two full ions in an electrolytic solution of monovalent ions. The cytoplasm in mammalian tissues is chiefly of monovalent cations such as K^+^ and Na^+^ balanced by monovalent anions such as Cl^−^ and HCO_3_^−^. The typical concentrations in humans and primates are about 150 mmol of each and give an osmotic total of ∼ 300 mmol per litre. In the steady state Debye and Huckel consider they are in complete electrostatic balance, each cation surrounded by anions and each anion surrounded by cations.

At the lower extreme of electrostatic forces are the induced temporary charges of weak Van der Waals forces that support non-polar hydrophilic hydrophobic interfaces. Strong Van der Waals forces can be formed by strong electrolytes and metallic ions. Moderate strength Van der Waals forces of the Debye type are formed with dipole molecules in the water interface [2].

Between 1974 and 1991 Widdas and Baker [3] applied the properties of the surface energy of water to explain the experimental findings on the penetration of sugars into, or out from, mammalian red cells. In the later glucose exit studies leading up to the theory in the 1991 paper, they used human erythrocytes, which had been obtained chiefly from their own blood samples, taken intravenously with implied consent. The saline medium was stirred in a cuvette with a 2 cm light path that was clear of the stirrer. The magnitude of light changes due to osmotic volume changes as sugars up to 100 mM entered or left the cells could be measured from tracings on a chart recorder. The special double-beam photoelectric technique had been developed in Widdas’ laboratory from 1950. This was shown to be reliable and quick by Sen and Widdas [4].

### 2.2. Sugar transport of human erythrocytes uses the surface energy of water

Experimental evidence suggested that surface tension of liquid water was the non-metabolic energy source for the linearly-inhibited rates of sugar exits by demonstrating reductions in the surface tension of the saline. This was confirmed by the finding that the half-saturation inhibitory concentrations for the four lower alcohols (methanol, ethanol, propanol and butanol) reduced threefold as this homologous series was ascended. This property, referred to as Traube’s Rule (1891), had been discovered by one of the late nineteenth century physical chemists (see Findlay, [5]).

The amino acid sequence of the human membrane transporter protein for glucose was described by nine biochemical investigators in 1985 (Mueckler *et al*. [6]). These authors described 12 alpha helices in the protein, which they suggested were embedded in the lipid membrane to provide the special transfer pathway for the water-soluble but lipid insoluble glucose molecule. After making a crude molecular model and studying possible mechanisms of working, it was decided that only the non-metabolic forces of surface tension of water, as roughly outlined, could effect the mechanical transport of sugar molecules through the red cell membrane. The scheme of membrane transport and the experimental evidence in its support were presented to the Physiological Society in two approved Communications given at the Bristol meeting of the Physiological Society in February 1990 [7, 8].

Despite the experimental evidence and the detailed scheme for muscle contraction given at the Bristol meeting, publication of a longer paper was rejected by two reputable Journals. The Editorial rejections included referees’ reports claiming that glucose binding could provide the energy of transport.

Ligand binding can easily be disproved by physical chemistry. Admittedly, binding can cause a mechanical pull effect on first binding if the induced or actual delta charges fit closely and are attracting. But electrostatic attractive forces, depending upon the inverse square of the distance, are maximal at zero separation. The strong attractions at minimal separation distances would have pulled the ligand to a fixed position and it would not release spontaneously. Therefore a cycle of relaxation and repeated attractions would be impossible without a source of energy to break the binding.

This elementary principle of physical chemistry is exemplified by the way that a kitchen timer with a magnetic strip adheres to the metal door of a domestic refrigerator with a loud adhesive ‘snap’ at zero distance. The timer does not fall off again without an applied force. Any scheme that provides for breaking the bonds of ligand binding to explain the transfer of millions of molecules by the process would require a second independent energy source. This is denied by those advocating a single energy source or simple ligand binding.

The total mechanical work in opening and closing a protein cleft by the glucose transporter protein can be determined from physical chemical principles since it involves the forceful breaking of water channels at both sides of the lipid membrane. The calculations described by Widdas [1] showed that the glucose protein transporters in five litres of blood, as representing all the red cells in the body, were doing 10 times more work per minute than was obtained from the resting metabolism of the adult.

This unexpected concept showed that the surface energy of water was capable of powering far more physiological mechanical work than thermal energy or any other cellular energy source. As stated on p22 of the 1993 paper [1]: “from the calculation, checked on the basis of the number of water molecules needed to fill the clefts (*ca*. 25 for each half-cycle), there is no escape from the conclusion that nature has found a way of using some of the thermal energy in cells to do work in membrane transport proteins in a manner that has never previously been suspected”.

Until this time, glucose transport had been regarded as passive since it always moved down a concentration gradient. Although the transport was seen to rely on a definite protein mechanism in the membrane region, it became described in the literature as “facilitated diffusion”. However, from the fact that the extra energy and work were without any additional metabolic requirement, other than that of the latent heat for evaporation taken from the surroundings, it was considered to be very unlikely that this free capacity to do work could have evolved solely for the glucose transporters in cell membranes. A greater widespread employment of this free work potential in physiological systems seemed to be more probable. Membrane protein transporters that are employed to transfer water soluble, but lipid insoluble, molecules into many bodily cells now form a family of proteins, all with similar functions. The family is termed Major Facilitator Superfamily (MFS) in the biochemical research laboratories that report on transporter proteins (see Abramson *et al*. [9]).

In a molecular scientific consideration of water molecules, the delta charges associated with the two hydrogen atoms of water (H_2_O) were shown to be remarkably powerful in attracting other water molecules. This was because of the separating angle of 105°: see Widdas and Baker [10, 11]. In the 2004 paper, significant proof of physiological applications was given in explaining the intermittence of patch clamp current changes as one of the mechanisms of working.

### 2.3. Experimental demonstration of surface tension effects in glass capillaries

Simple mathematical studies of the coherence of individual water molecules in the liquid surface demonstrate surface tension. Also, this is regularly seen in scientific laboratories that use teat pipettes. When a teat pipette is inserted into a beaker of water there is an immediate influx to form a column of water a few mm above the level of water in the beaker.

The physics is explained in physical chemical textbooks for biologists, such as Chang [12]. The downward force of the column of water is balanced by the up thrust of the surface tension acting around the periphery of the tube. Mathematical analysis shows that the height of the water column (h) varies with the reciprocal of the radius of the capillary, thus:

(2) $$\gamma \;\text{.2}\;\;\pi \;\;\text{r }=\text{ h}\text{.}\;\pi \;\;{\text{r}}^{2}$$

where γ is the surface tension and r the radius, hence

(3) h = 2. γ / r

The range of water pressures illustrated in this Figure is trivial, but can be seen to increase as the radius gets smaller. When this linear relationship is extrapolated to smaller sizes, the surface tension of water (a function of the surface energy) becomes important for movements in physiology and molecular biology. In mammalian tissues the water may contain salts or solutes with groups that can exert electrostatic attractions or repulsions.

### 2.4. The cohesion of media due to the attraction of salts and the due to attraction of water

The cytoplasm is in osmotic equilibrium with the extra-cellular saline of about 300 mosmol per litre. This is equivalent to 300 osmols in 1 m^3^ and each cation and each anion must occupy a volume of 5.5 × 10^−27^ m^3^. If the space is a box of equal sides, the side of the box would be about 1.8 × 10^−9^ m. This is conveniently seen as 1.8 nm.

The same calculation done for the water molecules at 55.5 mol/litre gives the side of the box (for water) as 3.1 × 10^−10^ m or 0.31 nm. There could be about 5.7 (say 6) water molecules between each ion of an electrolyte solution such as found in cytoplasm. Many cations and anions in the media have water shells surrounding them but the cohesive force will be reduced by the dielectric constant of water. A comparison of the electrostatic cohesive forces can be made and is relevant here.

Regarding sarcoplasm of muscle cells (300 mmol/litre) as chiefly the monovalent K^+^ and Cl^−^ ions of opposite charge, the attractive force for each pair will be:

(4) $$\text{Force }=\text{ 2}\text{.3}\;\times 10^{-28}/((3.24\;\times 10^{-18})\times 80)=9.2\times 10^{-13}{\text{Nm}}^{2}$$

In water, we visualise that the delta positive hydrogen of one water molecule will be attracted to the double delta negative oxygen of its neighbour. The approach shown theoretically and in molecular models can be as short as 1.1 times the radius of the oxygen atom (about 0.16 nm) and the effective dielectric constant may be similar to that of a protein (for instance benzene; ɛ = 4.7). Using 5 as an approximation for the dielectric constant, the cohesive force of a medium of pure water becomes:

(5) $$\text{Force }=6\text{.0}\times \;10^{-11}{\text{Nm}}^{2}$$

This value is 65 times greater than the electrostatic forces tending to pull together the pairs of ions making up the electrolytes in muscle sarcoplasm. Further, one litre of cytoplasm (300 mosmol) contains 1.8×10^23^ ions, whereas one litre of water holds 55.5 moles or 3.3 × 10^25^ molecules. So there are 185 more water molecules than ions. Allowing for the electrostatic attraction of delta charges to be eight times less, the overall cohesion by the water may be estimated to be about 1,500-fold greater than the cohesion by the ions.

The high cohesive force of water was described in a lecture in London by Faraday in 1846. This difference in favour of the surface tension of water is a further reason for considering the surface energy of water as a possible energy source for movements of molecular structures in mammals.

### 2.5. The chemical energy and heat from ATP hydrolysis

The theory that the chemical energy from ATP hydrolysis is the main energy source for tissues that rely on energy from metabolism has already been mentioned. Some aspects are worthy of consideration. From a physical chemical point of view, the hydrolysis of ATP is unique. This is because ATP has a special adsorption site on the myosin head (see page 396 of Stryer’s Textbook [13]) and as a consequence is “aimed” like a gun, and the lighter product of the ATP splitting (the P_i_) being ejected in a unique direction by the electrostatic impulse, which could open a distinct cleft in the protein during its vector “push”. The vector force is proportional to the product of charges (3 × 2 = 6) and inversely related to the square of the separating distance multiplied by the dielectric constant. The fall-off in force with the inverse square of the distance means that the force starts stronger for the initial pushing apart of stationary protein structures, but once started, can be kept going for about 3–5 nm before the algebraic integration of the force times distance, giving the total work done, is balanced by the energy available from ATP hydrolysis.

An aspect of interest for future physical chemical studies may be the way in which theoretical and molecular studies and readings have suggested a two dimensional hierarchical order of the magnitude of open and closing cleft movements in molecular science.

Firstly; the smallest membrane transport movements are the membrane transfers due to the rocking of protein alpha-helices about a fulcrum. This analogy of a child’s seesaw made of a plank of wood and a big stone was the scheme proposed for the glucose transporter by Widdas and Baker [3]. The same scheme might be applied to membrane permeability of ions as an alternative to that of a simple hydrophilic channel with water attracting side groups such as hydroxyl, amino or sulfhydril groups.

Secondly, movements of whole water molecules in protein matrices are considered to be possible over a distance an order of magnitude greater. These water movements are due to the uneven delta charges and the electrogenic properties of oxygen. The two hydrogen nuclei of water (H_2_O) at the bond angle of 105° induce the formation of attractive chains of molecules when Debye analysed saline structures by semi-classical quantum mechanics. However, these parallel threads were of short length due to the possible angular attractions distorting the linear tendency. These factors may be limiting the size of cleft closure to one or two nanometres and the cleft length to roughly that found in X-ray analyses such as those of Rayment *et al*. [14] and modern work by Sweeney and Houdusse [15].

The presence of parallel threads of water molecules with their individual attractions between consecutive pairs of atoms open up the possibility of amplification of mechanical effects. Thus in biophysics we can recognise parallel chains as increasing the force of single threads n-fold where n is the number of threads in the bundle. With water there is no amplification due to the length but there could be multiplication of effective voltages in tissues that have directed charge separations as for example in electric eels.

Philosophically, these two hierarchical movements may be considered as roughly limited to 1.5 nanometres and about 3 – 5 nanometres. The situation of the functional reactions possible in molecular science may increase with amplification where possible. However, without some rigidity (as from solid hydrocarbons in the trunks of trees in botany) there are no interesting mechanisms to be evolved in mammals. Only after the biochemical advances have been made to find proteins that could control insoluble crystal structures have functional movements of the third hierarchical order of magnitude become possible.

Amplification alone leads to masses of spongy material around rocks but with crystals of bone and crab shells the evolution of huge sizes has occurred. Philosophically, it is in this third order of hierarchical magnitudes that a plethora of complicated functions have developed for study in mammals and human volunteers. This third order is not further studied in this report as there are no large surface energy or surface tension shortages beyond the two hierarchical orders of magnitude available and used in mammals.. The third order surface energy contractions of a whole enclosing protein myoplasm to change a long elliptical structure to a compact spherical one was once thought to be a factor in contractions but this is never used in living systems.

Modern advanced X-ray studies have shown in great detail the ATP pocket in muscle proteins. In their paper on the motor mechanism of myosin V, Sweeney and Houdusse [15] show clearly the movement involved in a closed cleft from a muscle in rigour that changes to an open cleft after addition of ATP. See Figure 6 on p. 1836 of their paper. Unfortunately there is no indication of the size but from the view of ATP in the Figure it is assumed that the cleft opens more than 5 nm and may be 10 nm long.

This is far larger than the cleft presumed to be opened and shut repeatedly in the erythrocyte membrane transporter protein for glucose by Widdas and Baker [16]; see also molecular models described in Section 3. There the cleft from rocking of three alpha helices in the membrane protein is only ∼ 1.5 nm in length or about half the thickness of the lipid membrane. A cleft over 6 times as long would have different physical chemical properties and could result in a significant amplification of the mechanical work done by water adhesion in closing the cleft.

Further, the opening could not be done like a seesaw of lengths of α-helices over a sugar molecule as fulcrum, but would depend upon the greater energy such as that of the expanding vector force of ATP hydrolysis to open the cleft. The amplification of work output comes from the increased areas of the cleft walls attracted by the Van der Waals’ forces of the polar water molecules as they are extruded by the high Laplace pressures through the mouth of the cleft but at the same time are closing the cleft walls by the adhesive pull of the narrowing internal cleft water volume.

To pull the two sides of a cleft together we can imagine strings of nematic water molecules with the delta positive hydrogen attracted to a neighbour’s delta negative oxygen (lone pair). Several threads in parallel would add up. The cleft walls would have an area in muscle protein that is probably 12 times larger than that in the red cell glucose protein transporters, and the force available for cleft closure would be amplified. In this function the properties of water are peculiar since the parallel threads are of limited length due to the all round attractions of the bulk water.

Filaments of condensed water vapour, pure liquid free of solutes: have end-to-end cohesion similar to that described earlier, but only stable for short distances of the order of one to two nm. On the other hand the forces exerted by threads in parallel can be amplified by simply adding up the number of filaments. The cross-sectional area for one water molecule, πr^2^ = π × (0.155 × 10^−9^)^2^ = 7.5^−20^ m^2^ is the same as the end of a string of water molecules. For example if the walls of a cleft were roughly 2 nm by 3 nm or 6 nm^2^ there would be room for 250 parallel water chains to draw the walls of the cleft together and effect the closure.

Long chains of water molecules across the clefts will be adherent. Thus, each water molecule delta positive hydrogen of (0.26 electronic charge but + ve) will be attracted to its neighbour’s double delta-negative oxygen at a distance of about 1.1 × r, but reduced by the square of the distance, say (1.21 × r^2^). The effective attractive force is, therefore, about (2.0/1.21 = 1.65) delta charges squared. The repulsion of the two oxygen double charges is separated by two oxygen radii therefore ((2 × 2)/2^2^) = 1.0 delta charges squared. The repulsion of the two delta-positive hydrogen atoms are ((1 × 1)/(2r)^2^) = (¼ + ¼) Overall the attraction is (1.65 – 1.0 – 0.25 – 0.25)/r^2^ = 0.15/r^2^.

Widdas [1] gave the MKS units for full charges as F = 2.3 × 10^−28^ N m^2.^. F for delta charges can be roughly simplified to ¼ × ½ = 1/8 of the effect of monovalent full charges. The pull on each chain of water molecules is the same as that of the individual bonds and this can be seen to be F = ((1/8 × 0.15 × 2.3 × 10^−28^)/r^2^) = 1.8^−10^ N. The force in newtons (N), times the distance moved equals work in Joules. Thus, each water thread can do work when it helps to close a protein cleft. Let us suppose a pull of 2 nm would be sufficient to complete the closure. Then each string would do the work of 1.8^−10^ × 2^−9^ = 3.6^−19^ J. To sum these items of work 250 times would give an amplified force total of 9^−17^ J.

To make a comparison with the energy from ATP hydrolysis that is used to open the cleft described by Sweeney and Houdusse [15], is almost impossible, but a crude attempt could be made by a series of rough assumptions. Starting with the proposal that ATP hydrolysis opens just one cleft and that this single cleft is closed by the summated cohesion of these threads of water pulling the cleft walls together. This work of closure is done individually many times as the bulk water is extruded by the surface tension effect described earlier. The narrowing of the cleft would be due to the extrusion of the bulk contents from the mouth of the cleft and consequent reduction of volume. It is a property of water that its surface tension remains the same when the volume (e.g. of a sphere) reduces Thus, a very rough estimate might be made by treating the clefts as single molecular structures and giving them an imaginary molar value by multiplying by Avogadro’s number. When this is done the molar value becomes (9^−17^ × 6.02^23^) = 54,180,000 J = 54 MJ. This value is more than 400 times larger than the 31 kJ molar energy of ATP-splitting.

Admittedly, the Van der Waals bonds for hydrophobic proteins might be less than allowed for and other factors may make the estimate doubtful. Nevertheless, the magnitude of this rough value, like those for the cohesion of cytoplasm discussed earlier, bring the surface energy of water into the forefront for consideration of the energetics of protein movements in mammals. In particular, the area of muscle contraction calls for reconsideration in further study by muscle scientists. A fuller consideration and application of the First Law of Thermodynamics is appropriate here.

### 2.6. Joule’s experiments which founded the First Law of Thermodynamics

Let us begin with the history. Working in Physics in Manchester, Joule had found that electric motors and steam engines in the family brewery works were doing mechanical tasks that wasted some of the work being done as they raised the temperature of the cooling water. His experiments measured the amount of work done by each energy source. Joule found that the quantity of heat wasted depended linearly upon the quantity of work being done. This concept was not well received by the Establishment where the opinions were based on Lavoisier and Laplace. These scientists had held the view that separate mechanisms like electricity and steam engines had their own indestructible source of heat. Joule replied by showing that mechanical paddles turned in a water tank could raise the water temperature. The equivalence between measured mechanical work and heat energy was accepted by Helmholtz in Germany.

Although Joule’s views were not wholly accepted when first published, they are now regarded as the foundation for the First Law of Thermodynamics. From a chemical point of view the First Law of Thermodynamics was incorporated into mathematical and practical terms by arguably the greatest American scientist of the nineteenth century: Willard Gibbs. Gibbs was able to separate the molecular energy which can do work in biological systems from that part which is hidden and not able to do any chemical work. This hidden work potential is nevertheless presumed to exist and is termed entropy. The energy that can do work is called enthalpy. For a simple mental concept, the entropy or hidden energy may be looked upon as vibrations or rotations inside the atoms which do not affect neighbours to produce any movements that raise their temperature. Such hidden energy is presumed to increase linearly with the absolute temperature and the Helmholtz equation is written as:

(6) F = U−TS

where F is the energy available to do chemical work (Helmholtz Free Energy), U the total internal energy, T the absolute temperature and S the constant of proportionality for the non-available energy with temperature and given as “per degree Kelvin”, for entropy. Many chemical effects involve changes in reactant pressure or volume and Gibbs provided a more complete equation:

(7) G = U−TS+PV

where G is the available energy (Gibbs’ Free Energy, P the pressure and V the final volume). Gibbs extended his treatment of energy and showed how tables of energy changes in some reversible chemical reactions could be prepared. These tables of data have proved to be of enormous benefit for the analysis of physical chemical reactions that involve gases or other chemical equilibrium. Joule believed that whatever was driving the mechanism (in which mechanical work was done to overcome the friction and viscosity of the system) just gave the same quantity of heat output for the same amount of work done irrespective of the engine doing the work.

The earlier views that muscle contraction was derived from the energy of ATP hydrolysis when measured in a bomb calorimeter were mistaken. In muscle science approached from the biochemical point of view, there may be a throwback to the Lavoisier concept that was replaced by the Joule-Helmholtz concept of modern thermodynamics. In the decades of the early twentieth century, muscle contraction associated with oxygen metabolism has been regarded as directly due to energy from a motley of substances starting with glycolysis leading to lactic acid production, then phosphate bond energy, creatine phosphate, creatine phosphatase, formation of ATP by mitochondria enzymes and finally from ATP-hydrolysis, as the available energy currency alone. Bagshaw [17] described in detail how biochemical developments covered several decades of advances and these are not further considered in this review of proteins that directly lead to mechanical functions. Whatever, is driving muscle shortening, it is the mechanical work done against friction and viscosity that produces the extra thermal vibrations and liquid expansion in our thermometers to record the rise in temperature. Thus, it is the mechanical work that produces heat but this heat is mixed with chemical heat common to many metabolic reactions in the cells and not specific to any particular power source. This was clear from the First Law of Thermodynamics based on Joule’s nineteenth-century concepts.

Therefore the hydrolysis of ATP, which itself is associated with movements, should not be attributed to the chemical energy of any particular chemical species. The physical chemical properties of known energy sources might be used to give cycles of molecular movements that can be interpreted as a molecular engine, but muscle science still needs such an engine to convert an energy source into purposeful mechanical movements. This is clear in the advanced X-ray crystallographic studies reported by Sweeney and Houdusse [15], when carefully read.

The mechanical shortening of muscle and, more recently, the movements created by kinesin and the sodium pump, have been extensively studied – not only by muscle scientists but also by those studying the sodium pump and related topics. These movements are universally presumed to be powered by the chemical energy of ATP hydrolysis. Thus ATP’s hydrolysis and ability to do mechanical electrostatic work in biological tissues is worthy of reconsideration as an important application of the fuller understanding of the First Law of Thermodynamics in muscle science.

Starting with the view that ATP-splitting opens protein clefts generated by electrostatic forces of repulsion as the two products ADP and P_i_ separate, and that the surface energy of water closes the same clefts, it can be seen that the chemical energy of the ATP molecule is not required. The mechanical work occurs when the walls of the cleft are drawn together by coherent strings of water molecules – as previously described. We can see that the movements would do work and generate heat by the Joule-Helmholtz concepts of the First Law of Thermodynamics.

The time taken for the rapid extrusion of water from the cleft can be estimated by using Poiseuille’s formula as reported in 1990. When this was done for glucose exit a time of less than 1 μs was calculated for complete extrusion of all the cell water. This cleft closing rate would clearly be much too fast for the power stroke in muscle, or for the shortening rates of muscle twitches reported in the literature.

Therefore the proposed application of the surface energy of water may need the time-slowing property of a spring. Thus, we may understand a function for the coiled coil in the myosin head as described by Stryer in his textbook. The physical chemical principle required may be that of rapid charging of the biological spring and a reduced rate of energy release for the slower movement in the power stroke.

The plane water surface facing a vacuum would be like any air/water interface and allow evaporation of water molecules. In evaporation, water molecules extract the latent heat of evaporation from the surrounding bulk water. This latent heat is returned to the walls of the cleft as the water vapour condenses into liquid water by collision with polar groups or with the water molecules that have already condensed. However, the liquid water molecules formed from condensed water vapour must contain the hidden energy of latent heat. This energy is the input of energy for each evaporation/condensation cycle and was pointed out for the mechanism of working for the glucose transporter proteins in red cell membranes by Widdas and Baker [3].

The novel property of cellular water that makes the surface energy of water the better and obvious choice occurs where the liquid water is formed *de novo* from an evaporation/condensation cycle which is included by design in the mechanism. When this is done, the energy of evaporation derived from the bulk water is not from any specific chemical heat energy but from all the metabolic processes of the tissue. Thus the generated heat does not cause any increase in oxidative metabolism.

Nevertheless, the surface energy does not donate mechanical energy for nothing. It uses the input of energy just mentioned. This input of heat energy effectively comes from the overall heat of metabolism generated by the tissue in which the water space occurs. When the space is produced in the hydrophobic protein of the myosin head, it can be filled rapidly by condensing water molecules. When full and forming a single phase with bulk water, the surface energy is ready to produce a rapid extrusion of the water by the high Laplace pressure. The water space in turn is that produced by the hydrolysis of ATP and is an essential part of the overall scheme of contraction in muscle. This cooperation in function is where the two energy sources are to work together and can be said to be mutually interrelated in muscle contractions and related protein contractile activity – such as that by kinesin.

In this working hypothesis for a model with two energy sources, the surface energy does not work like a steam engine but employs the latent heat of evaporation when eliminating a *de novo* area of surface that was derived from condensed water vapour. Thus, in muscle both the heat source and heat sink appear to be at the same temperature.

### 2.7. The Fenn Effect in muscle contractions in the frog

Wallace O. Fenn in the early 1920s worked with A.V. Hill while he was on leave from America. He also conducted some independent experiments with the same apparatus in a basement of the house. Fenn made some important measurements that were published in the Journal of Physiology 1923–4 [18]. In this work he found that when the muscle shortened and did work there was an extra release of heat energy, an output over and above the heat of the isometric contraction as measured by improved thermocouples. This unexpected property was termed the Fenn Effect and is still being investigated eighty years later by advanced physiological studies with human volunteers [19].

If the shortening is delayed (by the cleft being held in an isometric state and then allowed to shorten further) the effect on the generated heat can be predicted from the Joule-Helmholtz concept of the First Law of Thermodynamics. But Joule’s work, then 70 years old, had not become part of the Establishment’s views at the time. The adherents to the single energy source hypothesis visualised that a spring was charged at the time of the isometric contraction without affecting the thermocouple readings. In this hypothesis, the extra heat of the Fenn Effect might have come from the uncoiling of the spring. However, Fenn [20] showed that this could not be the explanation.

In recent advanced experimental studies with human volunteers, Elder *et al*. [19] have measured the oxygen cost of thigh muscle in producing knee extensions. They measured electrical stimulation during isometric clamping and during free swinging of the lower limb. The results were analysed in detail and the conclusion was that they were “consistent with the view that oxygen cost of dynamic and isometric actions is determined by different circumstances of mechanical interactions between actin and myosin in the sarcomere, and that muscle recruitment has only a minor role”. Overall, studies like these call for a closer understanding of the First Law of Thermodynamics, as proposed earlier in this report.

In the two energy sources model, Widdas and Baker [21, 11] suggested that the electrostatic repulsion of the ATP hydrolysis products performed work of protein expansion in creating clefts. These were “push” effects and the “pull” effects were by the second energy source – whatever it may be. Consequently, perhaps for pedantic accuracy, it can be asserted that the heat that originates from the work done by ATP plays no part in the shortening contraction of muscles. The work of muscle shortening ultimately determines the physical strength and prowess of volunteers in training for some sports. But it is the second energy source which provides the mechanical work of contraction (the power stroke). Indeed, there is doubt from published investigations by other investigators as to whether the hydrolysis of ATP by myosin is related to muscle shortening at all (Weizsacker [22], Ostap [23]; see also Widdas [10]).

The novel views of this report may have some scientific support in these recent studies of the hidden oxygen cost of oxygen production in human volunteers performing dynamic and isometric exercises. Whether these studies are consistent with the theory of the work done by surface energy depends upon the effectiveness of evaporation/condensation and this, in turn, depends upon the latent heat of the bulk water for evaporation. The heat is derived from oxidative metabolism of all the bodily cells and its true origin is not available for experimental proof.

However, the work done by ATP hydrolysis (29.3 kJ/mol) is less than that estimated to activate the hydrolysis (35 kJ/mol) but the activation energy is not used itself. This is because the energy of ATP hydrolysis is a vector force whereas the latent heat of water used on condensation is larger but is not a vector.

Nevertheless, there are problems of starting the ATP-splitting to create the electrostatic repulsion forces for the mechanical work. The difference in electric charges means that the work from electrostatic forces must be considered. The three phosphate groups of ATP have four negative charges, which, as like charges, will repel one another to be maximally separated (as far as chemical bonds allow) but will still be under strain. If a fifth negative charge was forcibly introduced, the mutual strain would be greatly increased and may exceed the strength of the ADP-P covalent chemical bond and break it – thus starting the hydrolysis. It was this extra strain that Widdas and Baker [16, 11] proposed as the cause of ATP-splitting in muscle and not just the use of magnesium cations to neutralise the negative ATP charges. In the reactions of an ATPase the activation timing can be important but see later for the uses of magnesium.

The force of repulsion of the three ADP negative charges for the two on P_I_ is six times that between single electronic charges. When separated over the first 0.4 to 0.6 nm following the hydrolysis, the mechanical work done is 49 × 10^−21^ J. This is derived by integration and is about the same as the energy of the bond divided by Avogadro’s number (29.3 × 10^3^/(6.02 × 10^23^)) = 48.7 × 10^21^. It is estimated that introducing a fifth negative charge into the existing four negative charges would require 58 × 10^−21^ J (35 kJ/mol). This value is more than the energy released on hydrolysis but was considered to be the equivalent to the activation energy of a chemical reaction. The hidden latent energy in condensing water vapour is 41 kJ/mol so the molecules of liquid water that leave the surface on evaporation as molecules of water vapour could have kinetic energy in excess of that necessary for activation of ATP hydrolysis.

In a vacuum the chemical heat of ATP-splitting would all be used up by separating ADP and P_i_ by about 0.4 nm. However, in travelling through a protein matrix the P_i_ may acquire a proton to become singly charged. Further, allowing for a protein dielectric constant of about 5.0 the repulsive force would be reduced by a factor of 2 × 5 = 10 and in consequence the integrated force could separate protein groups to four nanometres on ATP-splitting. This fortuitous reduction of tenfold in the force brings the linkage of the energetics of water surface closures into line with the second order hierarchical opening by the vector force of ATP and gives a closer fit to the theme of the whole article.

Widdas and Baker [3] proposed that the negative charge to be introduced to the ATP was the negative hydroxyl anion from the dissociation of water. I proposed that bombarding molecules could transfer the impulse through several layers of water by the principles of shock transfers of energy in Newton’s Cradles. However, the shock transfer of energy through a semi-crystallised protein system of fixed water and other molecules of muscle cytoplasm is affected by the atomic structures in the pathways. The shock transfer through water is mainly slowed down by atoms of different mass from that of the water molecule (18 Da per mole).

Perry, in an 1897 engineering textbook, showed that on impact there is an interplay between the conservation of momentum and the conservation of kinetic energy. There is always a loss of transmitted energy if molecules of different mass from those of water are encountered. The loss is greater the larger the difference in mass. If the damping is not too great the condensation of water vapour could supply the energy necessary to trigger the hydrolysis of ATP.

On impact we can write:

**Table:** 

Conservation of Momentum	mv	(a vector)
Conservation of Kinetic Energy	½ mv^2^	(not a vector)

Shock transfer through bodies of equal mass:

$$\begin{array}{lll} \text{mv}\;\to & \text{mv},\text{mv},\text{mv},\text{mv}, & \to \;\text{mv} \end{array}$$

There is little change in v and hence in kinetic energy of the impacting molecule.

Shock transfer through bodies of equal mass have little change in velocity. If the velocity is unchanged, the kinetic energy of the projectile that is handed on by the impact to the packed molecules would also be nearly unchanged. Impacts involving different masses always reduce the kinetic energy of the projectile. Thus for two bodies of masses m_1_ and m_2_ the final velocity is given by:

(8) $$\text{Final v }=(2\;\times \;{\text{m}}_{1}/\;({\text{m}}_{1}/{\text{m}}_{2}\text{))}\;\times \;\text{intial v}$$

and there is always a reduction in the final kinetic energy.

When this work with the sodium pump was being considered, the reduction of the impact was estimated for the effect of three sodium ions in an occluded protein cleft ready to be extruded through the membrane. The sodium ions were replaced by other ions. The values for several ions were interesting in showing the effect of bodies of different mass.

Considering water and other possible ions:

**Table:** 

Ion	Li^+^	(H_2_O)	Na^+^	Mg^++^	K^+^	Rb^+^	Cs^+^	(HPO_4_^2−^)
Mol Wt.	7	(18)	23	24	39	85	133	96
Rel. K. E.	0.81	1.0	0.985	0.977	0.86	0.58	0.42	0.53
kJ/mol #	21.5	(41)	39.2	38.2	26.1	8.0	3.0	(21.7)

# The energy in kJ/mol represents the energy left if three of these ions were in the pathway for the impact.

The results show how the activation energy for ATP hydrolysis (35 kJ/mol) could be provided by the landing of condensing molecules on a region of packed cytoplasm at the ATP site. The landing molecules would have the hidden energy of latent heat and would transmit energy through any reasonable distance to start ATP-splitting and hence provide the mechanical work of opening a protein cleft. However, none of the ions shown would transmit enough energy to match the activation energy (35 kJ/mol) except for sodium and magnesium. These two cations are closely associated with the main users of the available energy currency in mammals, namely, ATP- splitting in muscles and other tissues studied in neuroscience, such as kinesin and carbonic anhydrase.

Muscle scientists use magnesium to ensure that ATP hydrolysis occurs, but their experiments are usually done on the bench [17] or with extracts from muscles that are interpreted to be complexes between two positive charges on the magnesium with two of the four negative charges of ATP Nath [24]. What causes the activation of ATP hydrolysis is never explained as the reaction is presumed to be enzymatic and associated with an ATPase or other enzyme. In my opinion the properties of water do not need to rely on the omnipotence of enzymes and physical chemical principles are usually sufficient.

### 2.8. The impact of modern reports in the fields of muscle and related science

The impact of recent publications and theoretical implications is worthy of consideration. Besides energetic considerations, the proposed working hypothesis for the two energy source model is helped by structural detail published on the Internet by K.C. Holmes [25]. His figures were based on structures derived from X-ray crystallography. One sentence from the legend to his figure 4 will be quoted in full. “For example one finds that the cleft in myosin extends from the ATP binding site to the actin binding site and that the opening and closing of this cleft is very likely to provide the communication between the ATP site and the actin binding site”. This structural detail indicates that that cleft may be the one opened by the vector force of ATP hydrolysis. The channel could be the pathway whereby the water extruded for the power stroke reaches the actin site and breaks the electrostatic bonds due to its high dielectric constant. This possibility immediately offers further facts for a working hypothesis for an “engine” to produce shortening, which may be worth considering by experts.

Stryer in his textbook has pointed out that actin is an ATPase but that the ATP splitting is not involved in the power stroke. Actin could, nevertheless, cause part of the shortening at each power stroke if the surface energy of water is involved. Assuming that actin monomers have a radius of 5 nm with linkages that hold them close together so that the separations are about 10 nm, bihemispherical gaps between the monomers would be much too small to admit liquid water. The cytoplasm would form a plane of saline across the tangents with a near vacuum in the separating spaces.

Evaporation across the interface would fill the “clefts” between the spheres but once full, the water would be extruded again to leave a vacuum. In this empty state electrostatic association of charged ions with slanting neighbours of the arrow formation described could bend each linkage to one side or the other. The forces would be large in the absence of water and tend to induce a sort of zigzag bending of the whole actin chain, which would shorten. The zigzag shortening would only need a bending of about 10 degrees in each of about 30 of 100 monomers to produce a shortening of 6 nm. The rapid extrusion of water with its high dielectric constant would release the bonds in the actin site and this would end the shortening until the next contraction. This is a purely hypothetical idea but illustrates a plausible mechanism that has the logical outlines of the “engine” that is necessary when employing two energy sources.

The sequential occurrence of actomyosin cycles may be different if only a single myosin complex is recorded - as in the advanced techniques with optical ‘tweezers’ as described and experimentally used by Veigel *et al.* [26]. These authors measured two contractions in rapid succession with a varying delay after the second. Their results suggested that two power strokes of about 6 nm occurred, not a single shortening of about 11 nm. It may thus be possible that actin shortens without ATP-splitting to make one contribution and that the myosin head mechanism provides the second contraction so that the two movements total about 11 nm, as described in Stryer’s textbook. Therefore, the side chains on the actin spheres may be linked by the activating divalent cation and the tension developed by the electrolytic zigzag formation. This theoretical possibility is an example of an “engine” for use in the two energy source model.

A recent seventeen-page article on the biochemical energetics of ATP energy was published by Nath [24] and contains many points relevant to this review. However, Nath presumes that the whole of the energy comes from the transduction of chemical energy into energy of movement or work. Topics that are advanced include the merits of coiling and uncoiling springs of coiled coil heptads of amino acid sequences in myosin, the binding energies of ADP and P_i_ when just part of an ATP complex with an enzyme, together with many other aspects of muscle science. However, there is no clear explanation of what acts as an engine. The physical chemistry of some of 16 lines will be discussed.

On page 2223 we find: “The propensity of the S-2 coiled coil to recoil is the thermodynamic driving force of the muscle power stroke. This recoiling of the of the S-2 N-terminal heptads leads to untilting and constrained rotation of the myosin heads bound to actin filaments which causes the power stroke exactly as detailed before”.

My difficulty is in understanding how “propensity” can become a driving force in thermodynamics or how uncoiling is energised and can lead to tilting or whatever is meant by “constrained rotation”, or how it could cause the power stroke.

Nath continues by stating that the “magnitude of the force generated by the elementary power stroke obtained with thermodynamic analysis is in perfect accord with both single molecule data as well as estimations of force generated by stage 2 insect flight muscle cross-bridges using 3D electron tomographic visualization. The mechanism also explains the valuable anti-S-2 antibody data of Sugi and Harrington. Above all, Nath’s rotation-uncoiling-tilt (RUT) energy storage mechanism of muscle contraction elucidates why myosin II possesses a double headed and double tailed structure, which had puzzled scientists for several decades” (The lines quoted were interspersed with numerical references to Nath’s other reprints not included here).

The energy of ATP as a molecule is envisaged to be stored as a torsion of the head groups of myosin due to partial unrolling of heptads of coiled coil spring-like structures in the head groups. The mechanical work done in the muscle contraction is argued as being due to changes in binding strength to the actin fibres during shortening. By placing reliance on binding energies changing from strong to weak and depending on ADP binding and that of P_i_, the scheme lacks any clear explanation as to what does the mechanical work effectively as an “engine”. The reliance on literary skills in place of experimental and logical evidence was used by Ling [27] in a previous decade. The theme of his approach was based on ATP inducing reorientation of fragments of protein with adsorptive properties that induced a different distribution of mobile ions in the medium.

### 2.9. Discussion of mechanical work energy derived from chemical energy

In this review it is proposed to limit the discussion of the functional interpretations of energy sources to the two different forms of mechanical energy. That of chemical energy derived from Adenosine Triphosphate (ATP) hydrolysis and that of the mechanical energy derived from the surface energy of water. The only comments will be based on the accepted view as expressed in the Encyclopaedia Britannica 1999 where it is stated that “Muscles use the free energy released by chemical reactions by coupling the chemical reaction to the physical changes in the contractile proteins”. Then, after disclaiming a complete understanding, the article goes on to state that “Of the reactions that have been identified, the splitting of ATP is the energy-yielding reaction nearest to the contractile event. Water participates in this reaction in which ATP is broken down into ADP and phosphate (P_i_): the reaction that occurs in the muscle, during which chemical free energy is converted into work can be written as follows:

$$\text{ATP }+\;{\text{H}}_{2}\text{O }+\;\text{contractile elements }\to \text{ADP }+{\text{ P}}_{\text{i}}\;+\;\text{contractile}\;\text{elements}\;+\;\text{work}\;+\;\text{heat}$$

This equation emphasizes the obligatory role of the contractile elements and the coupled nature of the reaction that produces work”.

Widdas and Baker [16, 11] proposed that the hydroxyl ion (OH^−^) and not water starts the ATP hydrolysis and that the work was done immediately by the electrostatic repulsion of the hydrolysis products. The 3 and 2 negative charges respectively, give a forceful repulsion proportional to their products i.e. 3 × 2 = 6, which opens a cleft at the ATP binding site. This mechanistic model does not invoke any magical properties on the part of contractile elements as implied by the equation from the Encyclopaedia, which is typical of many published ideas. See for example the opening paragraph in the ten author article by Rayment *et al.* [14]. Much of the biochemistry in the muscle system (such as that in Hogan’s laboratory) has been based on the parallel evolution of changes in metabolism that may appear to have been designed to replenish the essential role of ATP used by muscle cells during activity.

The investigating scientists who are interested in cellular movements can be roughly grouped into two camps. There is a group who have studied colloids and consider the importance of bound water in living cells. R.A. Gortner [28] and A. V. Hill took the main parts in a Symposium on “The state of water in colloidal and living systems.” held as a Discussion of the Faraday Society in 1930. The report of this meeting published as Faraday Society Proceedings is worth reading to understand how these different views have become polarised to give the present opinions generally favouring the A.V. Hill faction.

It is chiefly in elucidating the different pathways to provide the oxidative metabolic energy that A.V. Hill’s myothermic evidence has had its classical influence on the opinions of those in the muscle field. Thus, the current interest and advances in exercise and sports medicine is also based on this key position of ATP hydrolysis in the physiology and biochemistry of muscular activity. The overall effects on the oxygen metabolism of subjects exercising on bicycle ergometers are now popular fields of study where advances in knowledge of muscle physiology are being made. However, it should be realised that the provision of ATP hydrolysis is absolutely essential to the contraction mechanism in muscles by virtue of the vector force which ATP provides to do the cleft opening.

The heat energy produced by contractions in smooth muscle was studied ten years ago in Australia by Walker *et al*. [29]. They found that the amount of heat for maintaining tension in rat smooth muscle was reduced three fold after 10 minutes relative to the value at 10 seconds. This result also casts doubt on the use of ATP hydrolysis energy for the mechanical work of muscle contractions and heat production. Further this result might more easily fit in with the Joule-Helmholtz view that the heat production is proportional to the amount of mechanical work done irrespective of the mechanical source for the work. In muscle shortening essentially the same occurs as was shown by Fenn in his independent experiments reported in 1923 and now referred to as the Fenn Effect.

Muscle scientists have published a whole range of new ideas from the heat signals correlated with muscle twitches. The shift to the ideas of using ATP-hydrolysis as the only energy source necessary can be seen to have arisen with the biochemical advances from glycolysis and lactic acid, to creatine phosphate and oxidative metabolism in mitochondria, all as sources of ATP, which is now regarded as the main energy currency in the body of mammals. Changes in ATP concentrations in muscle occur during the contractile activity but these detailed biochemical advances leading to oxygen metabolism are outside this review.

After Engelhardt and Lyubimova showed that actomyosin was an ATPase in 1939, this compound (ATP) became the centre of investigation and Bernstein’s 1908 paper [30] in which he had advance surface energy as a factor in muscle contraction ceased to be referenced in muscle literature. The hiatus of the Second World War prevented muscle protein studies in many British and European laboratories. The centre of Interest in the single source of energy, namely that of ATP hydrolysis, has continued in the post-war era and no evidence to suggest that two energy sources may need to be considered came to light during the decades up to the beginning of the twenty-first century.

If two energy sources come to be considered in future, the surface energy of water is the obvious choice for the second energy source. Not only is it free of any need of extra oxygen metabolism, but its negative temperature coefficient, which increases muscle efficiency and strength by cooling towards freezing point, would be of distinct advantage to cold blooded animals and fish as well as to Canadian frogs and other amphibians acclimatised to living at subzero temperatures.

Cycles of evaporation and condensation followed by the use of the non-metabolic surface energy is an aspect of physical chemistry worthy of consideration at this late stage. Thus, actin monomers of radii about 3 to 6 nm will form rods of close packed spheres with gaps too small to accept liquid water of cytoplasm. They have minimal joining to keep them in line as a continuous rod of spheres. Cycles of evaporation/condensation, similar to those discussed in this review, would be set up across the flat planes in the cytoplasm over the tangents to the projecting spherical surfaces. These would cause local osmotic gradients as they formed a flat water surface with the composition of the bulk cytoplasm.

Microscopists would initially see near-distilled water being deposited into the empty clefts between the spheres. When the gaps are full and cleft water is able to mix as a single phase of liquid, the condensation of nearly pure water, free of salts and solutes, would create the necessary gradient with the bulk water interface. An osmotic water flow set up due to the concentration differences between local volumes at the two sides of the tangential plane could be another factor in the streaming of liquid that is seen under the microscope.

**Figure 1.**
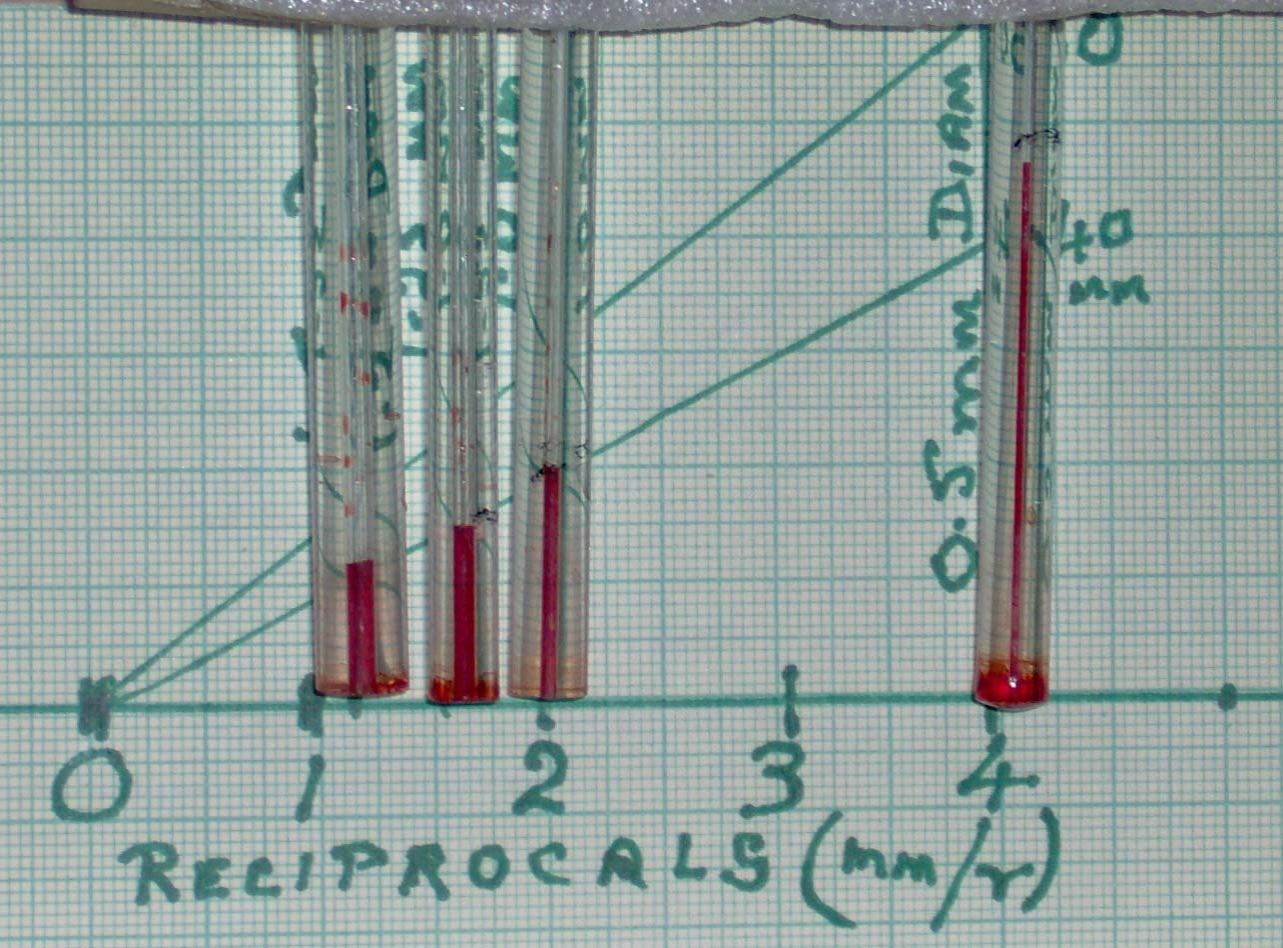
A group of narrow glass capillary tubes with columns of water drawn up to the balanced level are shown and the heights of the columns are seen to depend on the reciprocals of the radii. The exact heights may vary if the wetting angle is not along the lining of the tube [12, 14]. In the experimental set up here, on a table in a home laboratory in glass capillaries obtained as described briefly in Section 3, the water has been stained red to show up clearly in the photograph.

The application of energy sources to explain purposeful movements in muscle is a converging field of interest for scientific investigation. In this review, the surface energy of water has been shown theoretically to be important for the cohesive forces in water and saline media. Widdas [11, 30] has also shown surface energy to be involved in the origin of surface tension in tissues and illustrated in Figure 1. In extending the latter report, allowing for surface structural changes, Widdas [32] has suggested that the process of evaporation itself may be driven by electrostatic impulses. This may speed up the closures of the clefts opened by ATP hydrolysis. The modern work with human volunteers Elder *et al*. [19] supporting that of Fenn calls for the more complete understanding of thermodynamics and the Joule concept of heat generation.

Returning to muscle science, note that important results were published recently by Widen and Barclay [33]. These authors were able, by the use of improved experimental technology and careful protocols, to measure the forces of twitches in muscle samples and at the same time record the myothermic signals from sensitive thermocouples close to very small mouse papillary muscle preparations. In my interpretation of their results, they showed that the twitch force, measured by a capacity manometer and converted into energy by algebraic integration, was larger than the myothermal heat recorded. This result is what would be expected if the heat and mechanical work arose as described in the model discussed here. In other words the twitch would be due to the energy from the surface energy of water creating the power stroke. The force of contraction, which is converted into heat locally (the initial heat) would have no relation to the heat energy of ATP hydrolysis, but would be a measure of the violent disturbance of proteins close to the thermocouples and due to the rapid extrusion of water from the cleft at the time of contraction. The fact that, in these experiments, the initial heat from the thermocouple signal was recorded simultaneously with the twitch tends to support the suggestion of their common time origin. This cleft might be similar in principle to that of the model described in this review even if the cleft in cardiac muscle is structurally different from that in skeletal muscle.

Overall, it is considered that the scientific opinions regarding muscle physiology and biochemistry may take a new look at the surface energy of water first suggested by Bernstein a hundred years ago. Indeed, it is confidently anticipated that the subject of the surface energy of water will figure more prominently in reports of muscle science in the coming decades of the twenty-first century.

## 3. Experimental Section

A molecular model of the glucose membrane transporter was made using a length of electric flex (ca 0.25 m) to represent the 492 amino acid chain. The flex was inserted into twelve 10 cm lengths of acrylic tubing (outside diameter 12 mm) to represent the rigid alpha-helical intramembranous domains but was left free for the interdomain linkages. The scale of the model was roughly 10 cm i.e. 4 nm. This model showed that only three of the rigid lengths of alpha helices need to rock about a fulcrum provided by a spherical sugar molecule or water crystal of the same size, to effect an opening of nine alpha helices at one end and a closed flattened hexagon at the other end of a membrane protein Widdas and Baker [3].

To study the possible organisation of water in unstirred layers, molecules were made from plastic ball feet. To make models of water molecules, two holes were drilled at 105° into plastic spheres (r = 15.5 mm) representing oxygen atoms with a counterbore. Small white spheres were glued into the holes to represent the hydrogen bulges. To make crystal models of molecular “props” several water molecules were held together with fine polythene rods which were tight fits for holes drilled in the plastic spheres. The model sizes were considered adequate for illustration and rough estimates of the Coulomb forces.

Widdas and Baker [21, 11] made wooden perimeters of rectangular frames to contain a number of spherical ball feet used for molecular models. To show the minimum packing more clearly, only one side of the rectangle was fixed. The other three wooden sides (of the same length as in the rectangle) were hinged so that they could move to allow the shape to be changed from that of a rectangle to that of a parallelogram. The first rectangular model held 35 model spheres in a 5 × 7 array. In later models the mobile sides were made to hold 54 (6 × 9) and 25 (5 × 5) spheres. In addition, fixed examples of cuboid packing and hexagonal packing were prepared as described by Widdas [32]. These models were not capable of illustrating the details of the transformations that are possible but they gave an indication of their differences when the transformations had been made.

Digital photographs or two dimensional figures of these models are not as rewarding as their study in three dimensions. This is particularly clear in the close fitting of molecular models of water in the cuboidal/hexagonal transitions described by Widdas [32] and which, therefore, affect the appreciation possible from discussion in the text. Thus, the later position of this Experimental Section is well justified.

## 4. Conclusions

The principles of physical chemistry and mechanical logic can be applied to show how two energy sources may give a better coverage of the energetics of muscle contractions than a single energy source. Two energy sources may be converted into purposeful mechanical shortening in muscle fibres without invoking any magical properties of the protein constituents. It is not claimed that muscles work precisely as described, since there are myriad folds of structural detail to be included in a complete picture. Further, changed chemical and non-metabolic factors that may need modifications in detail are arising nearly every day.

The model described can give ATP-hydrolysis a logical mechanical part to play. This part uses the vector force to act as a repulsion to create a muscle cleft as a potential water channel. Indeed the vector force is an absolute essential to the “push” function of ATP hydrolysis in muscle. The key position of ATP hydrolysis in studies of muscle mechanics appears to have been associated with a parallel evolution of additional biochemical aspects of oxidative metabolism. One aspect is to maintain the running concentration of ATP available to the contracting muscle at a constant concentration level. This constancy of ATP concentration for the repetitive employment of ATP hydrolysis in muscle contractions is a further indication of the essential nature of the vector force of ATP hydrolysis, but it should be treated as a separate and biochemical aspect of muscle science.

In the energetic model, the actual force of shortening, the “pull”, is provided by a second energy source. The surface energy of water is that proposed for consideration. This form of energy, first suggested one hundred years ago but neglected for the last seven decades, is considered worthy of further study in the coming decades of the twenty-first century.
